# Assessing the Interplay between Weather and Septoria Leaf Blotch Severity on Lower Leaves on the Disease Risk on Upper Leaves in Winter Wheat

**DOI:** 10.3390/jof8111119

**Published:** 2022-10-24

**Authors:** Moussa El Jarroudi, Louis Kouadio, Jürgen Junk, Henri Maraite, Bernard Tychon, Philippe Delfosse

**Affiliations:** 1 Department of Environmental Sciences and Management, SPHERES Research Unit, University of Liège, 6700 Arlon, Belgium; 2 Centre for Applied Climate Sciences, University of Southern Queensland, Toowoomba, QLD 4350, Australia; 3 Environmental Research and Innovation, Luxembourg Institute of Science and Technology, 4422 Belvaux, Luxembourg; 4 Earth and Life Institute, Université Catholique de Louvain, 1348 Louvain-la-Neuve, Belgium; 5 Vice-Rectorate for Research, Maison du Savoir, Université de Luxembourg, 4365 Esch-sur-Alzette, Luxembourg

**Keywords:** *Zymoseptoria tritici*, plant disease risk, PROCULTURE, sustainability

## Abstract

Septoria leaf blotch (SLB) is among the most damaging foliar diseases of wheat worldwide. In this study, data for seven cropping seasons (2003–2009) at four representative wheat-growing sites in the Grand-Duchy of Luxembourg (GDL) were used to assess SLB risk on the three upper leaves (L3 to L1, L1 being the flag leaf) based on the combination of conducive weather conditions, simulated potential daily infection events by *Zymoseptoria tritici*, and SLB severity on lower leaves between stem elongation and mid-flowering. Results indicated that the variability in SLB severity on L3 to L1 at soft dough was significantly (*p* < 0.05) influenced by the disease severity on the lower leaf L5 at L3 emergence and the sum of daily mean air temperature between stem elongation and mid-flowering. Moreover, analyzing the predictive power of these variables through multiple linear regression indicated that the disease severity on L5 at L3 emergence and mild weather conditions between stem elongation and mid-flowering critically influenced the progress of SLB later in the season. Such results can help fine tune weather-based SLB risk models to guide optimal timing of fungicide application in winter wheat fields and ensure economic and ecological benefits.

## 1. Introduction

Severe epidemics of Septoria leaf blotch (SLB; causing pathogen *Zymoseptoria tritici*) can result in substantial yield losses in susceptible winter wheat cultivars if not well managed [1,2,3,4,5,6,7,8,9,10]. Such yield losses can reach up to 60% and negatively impact farm profits [11,12,13,14]. In countries such as France, Germany or the United Kingdom, potential financial losses could be more than €800 M nationwide [5,9]. In Belgium and the Grand-Duchy of Luxembourg, crop protection from fungal diseases relies largely on preventive fungicide application; hence winter wheat is routinely protected with two or three foliar treatments [15,16,17,18,19,20], with the aim of protecting upper leaf layers from disease epidemics given their critical contribution to the final grain yield [6,15,21,22,23]. With increased fungicide applications costs, increasing concerns for more environmentally friendly agricultural practices, and changes in the cost/revenue ratio for winter wheat, an accurate and reliable identification of optimum fungicide spraying is needed [23].

Various decision support systems (DSSs) are used to improve the control of fungal diseases in wheat [20,23,24,25,26,27]. For example, a DSS based on different plant disease models, which mathematically describe the relationships between the disease severity and conducive weather conditions to the main wheat fungal diseases (SLB, leaf rust, and stripe rust) in the Grand-Duchy of Luxembourg (GDL) [15,16,17,18], has been used over the past decade to determine whether fungicide use is needed, and if so, to guide the best application time for a single fungicide treatment [23]. The use of such DSSs in an operational context (i.e., guiding optimum time of fungicide applications in fields) requires a good knowledge on the interactions between the key factors favoring the onset and progress of the disease. Environmental factors such as rainfall, air temperature, relative air humidity, duration of leaf wetness, and inoculum concentration affect the development and progress of SLB in wheat [5,15,28,29,30,31,32,33,34,35,36]. Although the epidemiological development of SLB strongly depends on favorable meteorological conditions, differences of cropping practices also impact on the variability the onset, course, and severity of the disease from one site to another and from one year to the next [5,15,37,38,39]. Other factors include the uneven survival of pathogen inoculum and the uneven arrival of propagules from elsewhere [40]. The infections of lower leaves by either long distance spread of air-borne ascospores [41] or from those released from stubble of previous wheat plants occur routinely throughout winter and early spring in temperate wheat-growing regions [4,42,43].

Early infections of upper leaves most often require rain-splash dispersals onto the upper leaves of conidia produced on the lower leaves during stem-elongation [29,38]. Examples of Septoria leaf blotch risk models that integrate such spore movements within their framework is the PROCULTURE model [19,20,44]. Indeed, in PROCULTURE the leaf L3 (third leaf from the topmost leaf or flag leaf [L1]) can only be infected during its formation by the spores produced in lesions on either L5 or L4. L5 and L4 are low enough within the canopy and they can be infected by conidia produced by pycnidia from the bottom leaves [15,19]. Likewise, L2 can be infected only by symptomatic L4 or L3. However, understanding how the combined effects of weather and infection by *Z. tritici* early in the season inform the progress of SLB on the upper three leaves in winter wheat fields has yet to be fully investigated.

Thus, in this study we first aimed at assessing the risk of SLB development on the three upper leaves (L3 to L1) based on the combination of conducive weather conditions, potential infection events by *Z. tritici* and SLB severity on lower leaves between stem elongation and mid-flowering at four representative sites of the main cereal growing areas in the GDL. Secondly, the predictive power of the most important factors explaining the variability in SLB severity on the upper leaves was investigated. The identification of factors that favor SLB in winter wheat and their incorporation into plant disease risk models can help improve the performance of such models. This, in turn, can serve as a basis for developing strategies for timely and optimal fungicide applications according to the agro-climatological zone and disease occurrence and severity during the development of leaves that contribute the most to the final grain yield in winter wheat.

## 2. Materials and Methods

### 2.1. Experimental Fields Data and Disease Monitoring

To ensure the representativity of wheat growing areas across the GDL, the study fields were selected according to different criteria including the climate (representative of the two topoclimatological zones) and location (i.e., on a plateau, since winter wheat is cultivated primarily on plateaus in the GDL) [45]. Other criteria include grower’s farming experience and soil type. Thus, four sites were selected and monitored during the wheat growing seasons 2003 to 2009, with three sites (Burmerange, Christnach, and Everlange) located in the Gutland region (south of GDL) and one site (Reuler) located in the Oesling region (North of GDL). At each site and for each of the growing seasons, several wheat cultivars showing a range of susceptibility to SLB (Table 1) were sown in a randomized block design with four replicates (one replicate plot size = 8.0 m × 1.5 m). All crop management practices at the study sites (e.g., sowing and harvesting methods, weed and pest management) were similar to those in commercial fields in the GDL [15,46]. No fungicides were applied on plots evaluated during this study. A detailed description of the experimental method is found in [15,16]. Agronomic details for the study fields, as well as the dates of observation of L3 emergence, GS 59 (ear emergence complete), GS 65 (mid-flowering), and GS 85 (soft dough) [47] are presented in Table 1.

Hourly weather data collected from nearby (<2 km) automatic weather stations were used. Air temperatures (minimum, maximum, and mean) and relative air humidity were measured at a height of 2 m above the soil surface. Total rainfall was measured at 1 m above the soil surface.

During the study the disease incidence (proportion of plants with disease symptoms) and disease severity (percent leaf area diseased) were assessed on the same 10 plants in each plot (40 plants in total per cultivar and per site) throughout the monitoring period. Those plants were randomly selected and marked at the start of the monitoring. Disease assessments were made by experienced agronomists and trained raters weekly from April to July, with final observations at GS 77 (late milk) to GS 85 [47]. Prior to the disease monitoring, raters were trained using standard area diagrams [48] and disease assessment software (DISTRAIN [49]). Care was also taken to ensure the same rater assessed the same plot during each of the monitoring weeks [50,51,52].

### 2.2. Data Analyses

Eight variables related to weather conditions and disease severity at different crop growth stages were defined and used in this study. They were: (i) the percentage of SLB severity on L5 at L3 emergence (DS_L5_); (ii) the number of days with negative air temperatures during the period December 21 to March 21 (T_nw_); (iii) the number of days with rainfall ≥ 1 mm between GS 31 (first node detectable) and GS 65 (mid-flowering) (P1); (iv) the number of days with rainfall ≥ 5 mm between GS 31 and GS 65 (P5); (v) the total rainfall between GS 31 and GS 65 (ΣRain); (vi) the sum of daily mean air temperatures (base temperature = 0 °C) between GS 31 and GS 65 (ΣT); (vii) the number of potential daily infection events by *Z. tritici* between GS 31 and GS 65 (ID_PROC_(1)); and (viii) the number of simulated daily potential infection events greater than 1 between GS 31 and GS 65 (ID_PROC_(2)). The period between L3 emergence (GS 31) and mid-flowering (GS 65) was chosen as it is the most critical to the success of SLB control [16,21,23,38]. Potential daily infection events were simulated using the PROCULTURE model. PROCULTURE is a mechanistic disease model for SLB which simulates the potential infection events by *Z. tritici* and subsequent SLB epidemics in wheat under conducive favorable weather conditions, along with the development of the five youngest leaves L5 to L1 [15,29,44,54]. It uses hourly data of relative air humidity, air temperature, and rainfall sums, along with information on crop phenology during the season (e.g., observed date of L3 emergence) as input variables. It has been validated in the GDL [15].

Correlations between each of the defined variables and the average disease severity on the upper leaves at GS 85 were used to characterize the disease severity for each site. Then, a stepwise regression analysis was carried out to identify the top variables explaining the variability of SLB severity on the upper leaves at GS 85, and the prediction accuracy of the model was assessed through a leave-one-out cross-validation (LOOCV). LOOCV involves taking N-1 of the data points to build the model and testing the results against the remaining single data point, in N systematic way replicates, with the kth point being dropped in the kth replicate.

Several statistical indicators including the adjusted R^2^ (Adj. R^2^), the root mean square error (RMSE), and the Mallows’ Cp [55] were used to assess the performance of the models. Adjusted R^2^ is a measure which attempts to reduce the inflation in R^2^ by considering the number of independent variables and the number of cases. It was calculated as follows:

(1) $$\mathrm{Adj}.{\;R}^{2}\;=\;1\;-\;\frac{\left(n\;-\;1\right)\left(1\;-\;R^{2}\right)}{n\;-\;p}$$

where *n* is the total number of observations, and *p* is the number of predictors in the model.

The weighted variations in errors (residual) between the predicted and observed disease severity was given by the RMSE. It is one of the most widely used error measures and was calculated as follows:

(2) $$\mathrm{RMSE}\;=\;\sqrt{\frac{\mathrm{SSE}}{n\;-\;p}}$$

where SSE is the sum of square error.

Mallows’ Cp was used as a criterion for goodness-of-fit of regression equations. Acceptable models (i.e., those which minimize the total bias of the predicted values) are those with Cp values approaching the number of model’s parameters. The Mallows’ Cp was calculated as follows:

(3) $$C_{p}\;=\;\frac{{\mathrm{RSS}}_{p}}{{\hat{\sigma }}^{2}}\;-\;n\;+\;2p$$

where RSS is the residual sum of square of the submodel (with *p* parameters).

We also used the variance inflation factor (VIF) to check for the precision and stability of the model coefficient estimates. The VIF for a given predictor was calculated as follows:

(4) $${\mathrm{VIF}}_{i}\;=\;\frac{1}{1\;-\;R_{i}^{2}}$$

where $R_{i}^{2}$ is the R^2^ value obtained by regressing the ith predictor on the remaining predictors. A low VIF (<4) indicates good and stable coefficient estimates. there is no correlation among the ith predictor and the remaining predictor variables; a VIF > 4 is indicative of poorly estimates of regression coefficients [56].

All statistical analyses (analysis of variance (ANOVA) and stepwise regression) were performed using the general linear modelling and regression procedures of the software SAS^®^ (version 9.1; SAS Institute Inc., Cary, NC). In the ANOVA, the average percentage of infected leaf area of L3, L2, and L1 was used as dependent variable; the sites, cultivars and years were used as independent variables. Post-hoc tests (Tukey’s test) were carried out and *p*-values below 0.05 (2-sided) were considered as statistically significant.

## 3. Results

### 3.1. Septoria Leaf Blotch Severity during the Study Period

**Figure 1:**
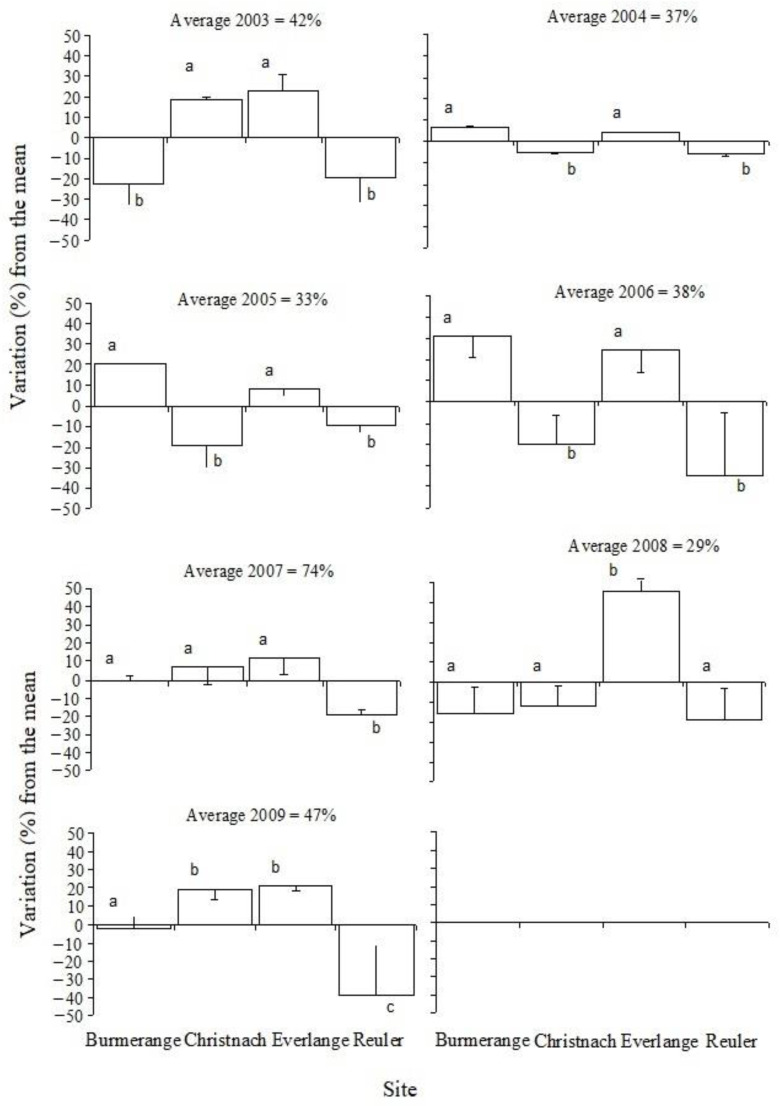
Variations of Septoria leaf blotch severity on the three upper leaves at soft dough (GS 85) for each site during the 2003–2009 period. Annual disease severity averages for all the sites. Statistically significant differences between sites (α = 0.05) are indicated by different letters.

The severity of SLB was high in the Gutland region (Burmerange, Christnach, and Everlange) and moderate in the Oesling region (Reuler) (Table 2). In the Gutland, SLB was more severe in the western part (Everlange) than in the eastern (Christnach) and southern (Burmerange) parts. At Everlange, the years with high SLB severity were 2007, 2008 and 2009, whereas they were 2006 and 2007 at Burmerange and 2007 and 2009 at Christnach. On the other hand, at Reuler, only 2007 was the year with the highest disease severity during the 7-year monitoring (Table 2). When comparing the mean annual disease severity at GS 85 among sites (Figure 1), SLB severity most often was higher at sites located in the Gutland, namely at Everlange, than that in the Oesling region. The ANOVA shown statistically significant differences among cultivars, sites, and years, as well as significant interaction between years and sites (*p* < 0.001). Such statistical differences can be explained, to some extent, by the differences in meteorological conditions between years and sites, as it is discussed in the next sections.

### 3.2. Weather Factors Influencing SLB Severity at The study Sites

**Figure 2:**
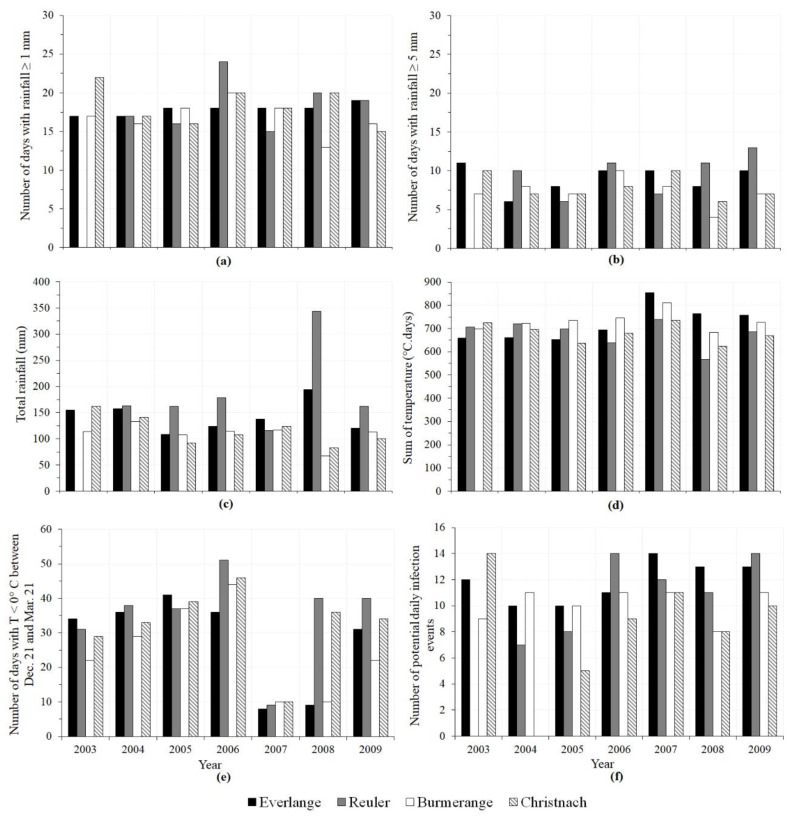
Interannual variations of rainfall and temperature conditions for each of the study sites during the 2003–2009 period. (**a**) Total number of days with rainfall ≥ 1 mm between GS 31 (first node detectable) and GS 65 (mid-flowering); (**b**) total number of days with rainfall ≥ 5 mm between GS 31 and GS 65; (**c**) total rainfall between GS 31 and GS 65; (**d**) cumulative sum of daily mean air temperatures (base temperature = 0 °C) between GS 31 and GS 65; (**e**) the number of days with negative air temperatures during the period December 21 to March 21; (**f**) number of potential daily infection events by *Zymoseptoria tritici* between GS 31 and GS 65.

Rainfall patterns varied from one site to another between L3 emergence (GS 31) and mid-flowering (GS 65) during the 2003 to 2009 cropping seasons (Figure 2a–c). No rainfall was recorded in 2003 at Reuler between GS 31 and GS 65 (Figure 2c), whereas in 2008 an exceptional high rainfall amount (343 mm) was recorded at this site during the same period (Figure 2c). During the same period, the number of days with rainfall ≥ 1 mm ranged from 17 ± 2 to 18 ± 2 in Christnach, Everlange, and Burmerange (Figure 2a). For a period spanning 1.5 to 2 months (Table 1) that means there was a rainy event almost every three to four days. At Reuler, this number was slightly high (19 ± 3) when considering years with rainfall recorded between GS 31 and GS 65. Regarding temperature, the cumulative temperature between GS 31 and GS 65 (ΣT) was above 600 °C.days for all study sites (Figure 2d). Moreover, in the year with highest SLB severity (2007), the December 21–March 21 period recorded the lowest (≤10) number of days with negative mean air temperatures in all sites (Figure 2e). The combination of such varying weather conditions between years for a given site, and between sites, was captured through the ANOVA (Table 2). For example, at Everlange where the same cultivar was sown during the study period, the average SLB severity observed on the three upper leaves at GS 85 in 2007 was statistically different (*p* < 0.05) from the average severity values in other years (Table 2). In 2004 and 2005 or 2003 and 2006, however, the disease severities observed were similar (*p* > 0.05), implying that the patterns of weather conditions during those years resulted in similar disease severity.

When analyzing the correlations between each potential explanatory variable and the average percentage of SLB severity on the three upper leaves L3 to L1, results indicated that, generally, ΣT was significantly correlated with the average percentage of SLB severity at all sites (*p* < 0.01 for Burmerange and Everlange; and *p* < 0.05 for Christnach and Reuler; Table 3). For T_nw_, its association with average SLB severity on the three upper leaves was statistically significant (*p* < 0.05) in most sites. Thus, for Everlange, Christnach and Reuler, the higher the number of days with negative mean air temperatures during December 21–March 21, the lower SLB severity on the upper three leaves later in the season (Table 3). At Burmerange, there was a different pattern with a weak correlation found (Table 3). Rainfall totals between L3 emergence and GS 65 (ΣRain) were not significantly correlated to SLB severity on the three upper leaves at GS 85 in most of the study sites (Table 3). Burmerange was the exception. In Everlange in 2007 (year with highest SLB severity) ΣRain was 137 mm, while in 2004 (year with lowest disease severity) ΣRain was 158 mm.

### 3.3. Relationship between Disease Severity on the Upper Leaves at GS 85 and Disease Severity on L5 at L3 Emergence and Simulated Potential Daily Infection Events

**Figure 3:**
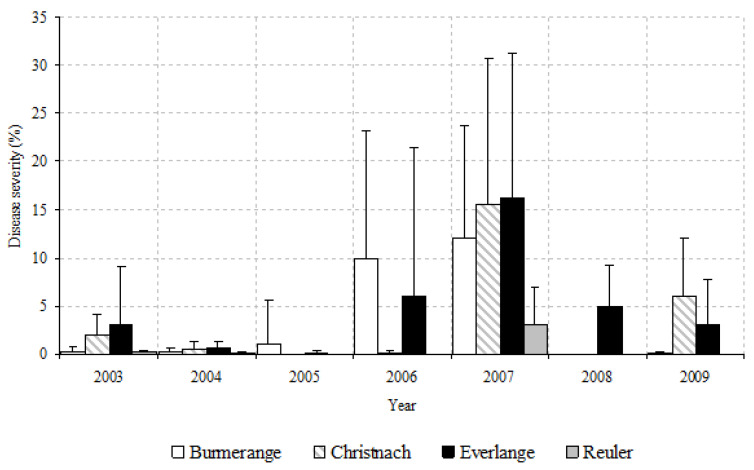
Average Septoria leaf blotch severity on leaf L5 (leaf L1 being the flag leaf) at the time of leaf L3 emergence during the cropping seasons 2003 to 2009. Bars indicate the standard errors.

There were noticeable year-to-year variations in SLB severity on L5 at the emergence of L3 (GS 31) over the 2003–2009 period (Figure 3). Regardless of the site, 2007 was the year with highest SLB severities on L5 at GS 31. Severity of SLB on L5 at GS 31 was significantly correlated to the disease severity on the upper three leaves at GS 85 (Table 3).

Positive and statistically significant associations (*p* < 0.05) between the number of potential infection days simulated using PROCULTURE and the average disease severity on the three upper leaves at GS 85 were found for Everlange, Christnach, and Burmerange during the study period. At Reuler there was no clear trend, resulting in a non-significant association. Indeed, the number of simulated potential infection days between L3 emergence and GS 65 in 2006 and 2009 was 14 days, while SLB severity on the upper leaves at GS 85 was low (3% and 8%, respectively). Whilst in 2007 when such disease severity at GS 85 was high (55%), the simulated potential infection days was only 12.

### 3.4. Modeling SLB Risk Severity

**Figure 4:**
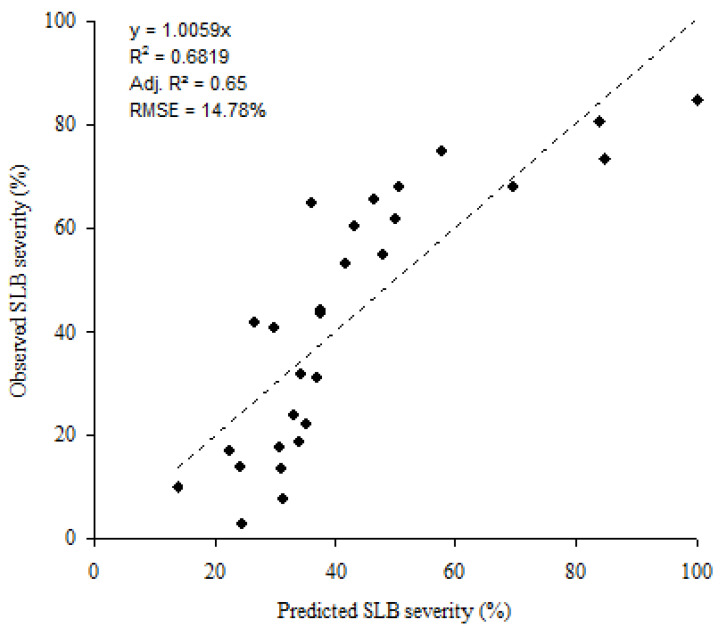
Scatterplot of predicted versus observed Septoria leaf blotch (SLB) at GS 85. Predicted SLB severity were performed using the most influencing variables for all sites and cropping seasons through multiple linear regression model.

A correlation analysis using the pooled data showed that DS_L5_, ΣT, ID_PROC_(1), and T_nw_ were significantly correlated (*p* < 0.05) to SLB severity on the three upper leaves at GS 85. Results of the stepwise regression modelling indicated that only DS_L5_ (SLB severity at L3 emergence) and ΣT (sum of daily mean air temperature between GS 31 and GS 65) substantially contributed to explaining the variability in SLB severity on the three upper leaves at GS 85. The two variables explained approximately 68% of the variability in SLB severity (Adj. R^2^ = 0.65), with a RMSE = 14.78% (Figure 4). The disease severity on the three upper leaves at GS 85 (Y) can be expressed by:

(5) $$Y\;=\;69.8\;+\;3{\;*\;\mathrm{DS}}_{L5}\;+\;0.15\;*\;\Sigma T$$

The regression-based model found was statistically significant (*p =* 0.03); the Mallows’ Cp was low (0.04). There was no multicollinearity between the selected explanatory variables with the VIF equaled 1.75, which indicates that the coefficients were properly estimated and stable.

The bootstrap analysis confirmed the satisfactory ability of the selected variables DSL5 and ΣT to predict the disease risk on the three upper leaves. The model was highly significant (*p* < 0.001). The LOOCV results showed that the LOOCV-R^2^ was 0.60 and the RMSE = 13.66. The lower and upper 95% confidence limits equaled 10.67 and 16.64, respectively. Moreover, the standard deviation of the mean of residuals found in the regression step was close (13.75) to LOOCV-RMSE.

## 4. Discussion

We investigated the relationships between Septoria leaf blotch severity on the three upper leaves L3 to L1 (L1 being the flag leaf) at soft dough (GS 85), weather conditions and SLB severity on lower leaves between stem elongation and mid-flowering in the Grand-Duchy of Luxembourg. During the 2003 to 2009 wheat cropping seasons SLB severity varied in the study sites. Such differences were explained by the combination of several environmental conditions. Several authors reported a negative correlation between the frequency of days with negative air temperature during winter (December–March) and SLB severity on the two upper leaves later in the season [21,32,57,58,59]. Our conclusions are in line with these studies, with statistically significant negative correlations (*p* < 0.05) found between the number of days with T < 0 °C during the period 21 December–21 March and the average disease severity on the three upper leaves in spring and early summer. The consequences of a mild winter on SBL progress later in the season became apparent in our study during the 2006/2007 cropping season with high SLB severities recorded at all locations. This relationship, demonstrated across a wide range of sites, seasons and cultivars, confirms the results previously reported from a selected data set of unsprayed crops of a single cultivar [59] and suggests that the potential for disease development might be quantifiable early in the season.

The importance of rain for SLB severity has been well documented [15,29,30,35,54,60,61]. Our results showed that rainfall totals between L3 emergence and GS 65 did not noticeably explain variation in SLB severity later in the season (GS 85). With sporadic rainfall, two crops differing in development by only two or three days can have very different disease levels [38]. Thus in PROCULTURE rainfall of 0.1 to 0.5 mm are also considered for infection and conidia splash and used in the simulation of SLB progress and development [15]. The strongest disease risk to a crop is the occurrence of conditions allowing spore transport while the three upper leaves are emerging.

At Reuler, the presence of the Ardennes Forest as natural barrier to the movement of spores (dominant western wind), and the higher number of days with negative winter temperatures could have influenced the severity on lower leaves in the spring. This may explain why for some years (i.e., 2006 and 2009) at this site, the number of simulated potential daily infection events outputted from PROCULTURE was high, but the degree of disease severity was low. Typically in early spring, if spores are fairly abundant, their numbers will be controlled largely by the time elapsed since crop emergence, which will determine how many generations have occurred and the cumulative green leaf area exposed during this time [38]. This cycle would be sufficient to maintain the fungal population in the crop and result in future infections by the pathogen.

In our analysis, the linear model was built based on the disease severity at L3 emergence (DS_L5_) and the sum of daily mean air temperature between GS 31 and GS 65 (ΣT). Potential interactions between climate variables, e.g., the distribution of defined periods of time with favorable weather conditions conducive to an infection event (i.e., relative air humidity > 60% during the 16h following a rainfall event and air temperature > 4 °C during the first four hours following such rainfall event [19], were not explicitly investigated. This was assumed to be implicitly considered through the simulated potential daily infection events using PROCULTURE. Including such interactions as explanatory variables could potentially improve the model performance.

Our study considered whether the combined effects of weather and SLB infection on L5 early in the season could predict the severity of SLB on the upper three leaves at GS 85 in winter wheat. The disease severity on L5 when L3 was emerging can partially explain the variation of the SLB epidemic among years during the study period. The severity on L5 at the beginning of L3 emergence thus appears to be an indicator of the future SLB severity on the last three leaves at GS 85. The model found indicates that the percentage of SLB severity on L5 at L3 emergence and the sum of daily mean air temperature between L3 emergence and GS 65 can be used to explain the severity of SLB on the three upper leaves at a later growth stage. Although the predictive power of the model developed in this study is better at high severity (severity ≥ 40%; Figure 3), suggesting the potential for using such piece of information to define thresholds for fungicide spraying. This can be part of future research. Future research can also include investigating the building-up of *Z. tritici* inoculum in the crop prior to GS 31 and its effects on SLB development later in the season, which was not carried out in this study. Shaw and Royle [38] reported that the risk of SLB progression on the upper leaves was dependent on the abundance of *Z. tritici* inoculum on the lower leaves. This could help improve the overall disease risk modelling and early disease warning system in the GDL.

## 5. Conclusions

The first step in the formulation of a plant disease management strategy is to identify the most important risk factors conducive to damaging epidemics. We assessed the relationships between Septoria leaf blotch severity on the three upper leaves, the disease severity on the lower leaf L5 at L3 emergence and simulated potential daily infection events by *Z. tritici* and weather conditions (i.e., total rainfall, number of days with rainfall ≥ 1 mm, number of days with rainfall ≥ 5 mm, sum of daily mean air temperatures) between stem elongation and mid-flowering, and the number of days with negative air temperatures during the period 21 December to 21 March at four representative wheat-growing sites in the Grand-Duchy of Luxembourg. Two variables, SLB severity on L5 at L3 emergence and the sum of daily mean air temperatures between GS 31 and GS 65, were found to be those explaining the most the variability in the disease severity on the three upper leaves at GS 85, with good predictive power of the linear regression model based on their combination. The model developed is simple as it depends on two variables easy to record or calculate. However, one of the main drawbacks of empirical models is that their application is often valid only for the area they have been calibrated for. Nevertheless, our study could contribute to fine-tune the optimal timing for fungicide application. Indeed, models which simulate the date of emergence and the development of the five youngest wheat leaves, as well as *Z. tritici* inoculum available to infect those leaves could be advantageously fine-tuned while integrating the degree of SLB severity on lower leaves (i.e., L5) at the start of L3 emergence. Decision-support tools based on in-season disease monitoring and weather-based disease models can guide the control of diseases epidemics while limiting potentially harmful side effects of excessive fungicide applications and ensuring economic benefits.

## Figures and Tables

**Table 1:** Agronomic information for fields of winter wheat at four experimental sites in the Grand-Duchy of Luxembourg during the cropping seasons 2003 to 2009.

Site	Region	Year	Sowing Date	Cultivar	SLB Susceptibility ^a^	Date of L3 Emergence	Date of GS 59 ^b^	Date of GS 65 ^b^	Date of GS 85 ^b^	Harvest Date
Burmerange	Gutland	2003	4 Oct. 2002	Dekan	4	14 Apr.	28 May	2 Jun.	23 Jun.	11 Jul.
(50°3′ N, 6°1′ E)		2004	1 Oct. 2003	Cubus	6	18 Apr.	1 Jun.	12 Jun.	5 Jul.	2 Aug.
		2005	13 Oct. 2004	Cubus	6	15 Apr.	3 Jun	6 Jun.	4 Jul.	4 Aug.
		2006	30 Sep. 2005	Cubus	6	15 Apr.	30 May	12 Jun.	3 Jul.	19 Jul.
		2007	11 Oct. 2006	Cubus	6	3 Apr.	16 May	23 May	18 Jun.	26 Jul.
		2008	6 Oct. 2007	Cubus	6	19 Apr.	30 May	4 Jun.	23 Jun.	5 Aug.
		2009	6 Oct. 2008	Cubus	6	17 Apr.	30 May	5 Jun.	29 Jun.	29 Jul.
Christnach	Gutland	2003	2 Oct. 2002	Flair	4	22 Apr.	5 Jun.	10 Jun.	7 Jul.	23 Jul.
(49°45′ N, 6°14′ E)		2004	13 Oct. 2003	Flair	4	23 Apr.	10 Jun.	22 Jun.	12 Jul.	12 Aug.
		2005	27 Oct. 2004	Rosario	5	25 Apr.	16 Jun.	20 Jun.	4 Jul.	2 Aug.
		2006	12 Oct. 2005	Flair	4	25 Apr.	12 Jun.	15 Jun.	3 Jul.	25 Jul.
		2007	12 Oct. 2006	Tommi	4	11 Apr.	21 May	1 Jun.	25 Jun.	26 Jul.
		2008	23 Oct. 2007	Flair	4	30 Apr.	4 Jun.	9 Jun.	7 Jul.	5 Aug.
		2009	23 Oct. 2008	Boomer	5	27 Apr.	2 Jun.	11 Jun.	6 Jul.	7 Aug.
Everlange	Gutland	2003	4 Oct. 2002	Achat	5	23 Apr.	2 Jun.	8 Jun.	30 Jun.	19 Jul.
(49°29′ N, 6°19′ E)		2004	14 Oct. 2003	Achat	5	19 Apr.	9 Jun.	14 Jun.	12 Jul.	6 Aug.
		2005	22 Oct. 2004	Achat	5	21 Apr.	8 Jun.	13 Jun.	4 Jul.	2 Aug.
		2006	10 Oct. 2005	Achat	5	23 Apr.	8 Jun.	12 Jun.	3 Jul.	7 Aug.
		2007	10 Oct. 2006	Achat	5	4 Apr.	21 May	2 Jun.	2 Jul.	26 Jul.
		2008	8 Oct. 2007	Achat	5	19 Apr.	2 Jun.	9 Jun.	7 Jul.	5 Aug.
		2009	13 Oct. 2008	Achat	5	20 Apr.	4 Jun.	8 Jun.	6 Jul.	6 Aug.
Reuler	Oesling	2003	6 Nov. 2002	Bussard	6	30 Apr.	12 Jun.	16 Jun.	7 Jul.	5 Aug.
(50°11′ N, 5°15′ E)		2004	16 Oct. 2003	Bussard	6	26 Apr.	16 Jun.	22 Jun.	12 Jul.	16 Aug.
		2005	5 Oct. 2004	Flair	4	25 Apr.	16 Jun.	20 Jun.	4 Jul.	13 Aug.
		2006	13 Oct. 2005	Dekan	4	4 May	14 Jun.	19 Jun.	3 Jul.	8 Aug.
		2007	7 Oct. 2006	Akteur	6	15 Apr.	29 May	6 Jun.	25 Jun.	3 Aug.
		2008	10 Oct. 2007	Schamane	4	9 May	8 Jun.	12 Jun.	7 Jul.	14 Aug.
		2009	10 Oct. 2008	Schamane	4	30 Apr.	15 Jun.	21 Jun.	6 Jul.	18 Aug.

^a^ SLB susceptibility: scale 1 (low susceptibility) to 9 (high susceptibility) [53]. ^b^ Growth stage (GS) 59: Ear emergence complete; GS 65: Anthesis half-way (anthers occurring half way to tip and base of ear); GS 85: Soft dough.

**Table 2:** Visually estimated leaf area (%) covered by *Zymoseptoria tritici* lesions at soft dough (growth stage 85) at the study sites during the cropping seasons 2003 to 2009. Annual mean values of the disease severity on the upper leaves L3 to L1 (L1 being the flag leaf) and their standard deviations (SD; expressed in percentage) are provided. For a given site, statistically different means are indicated by different letters (level of significance α = 0.05).

	Burmerange		Christnach		Everlange		Reuler	
Cropping Season	Mean	SD	Mean	SD	Mean	SD	Mean	SD
2003	19.0 c	21.4	60.6 b	30.7	64.5 c	23.5	22.5 c	19.5
2004	43.7 b	29.7	31.9 c	29.4	41.2 d	30.6	31.2 b	29.3
2005	53.1 b	30.7	14.1 d	18.3	41.7 d	25.1	23.8 bc	25.6
2006	68.3 a	25.1	17.8 d	21.0	62.2 c	24.1	3.2 d	4.8
2007	73.5 a	28.2	80.9 a	16.2	85.1 a	17.2	55.1 a	27.7
2008	13.6 c	22.7	17.2 d	24.7	74.7 b	24.6	10.1 d	19.8
2009	44.2 b	29.9	65.7 b	31.3	68.2 bc	32.9	8.0 d	9.8

**Table 3:** Pearson correlation coefficients between the average Septoria leaf blotch severity on the three upper leaves (L3 to L1, L1 being the flag leaf) at GS 85 and selected variables for each of the study Luxembourgish study sites.

Site	Variable ^a^	R	Prob > |R| under H0: Rho = 0 ^b^
Everlange	DS_L5_	0.82	*
	T_nw_	−0.83	*
	ID_PROC_(1)	0.96	***
	P1	0.37	ns
	P5	0.62	ns
	ΣRain	0.07	ns
	ΣT	0.75	*
	ID_PROC_(2)	0.42	ns
Burmerange	DS_L5_	0.80	*
	T_nw_	0.37	ns
	ID_PROC_(1)	0.84	*
	P1	0.78	*
	P5	0.78	*
	ΣRain	0.53	ns
	ΣT	0.91	**
	ID_PROC_(2)	0.50	ns
Christnach	DS_L5_	0.84	*
	T_nw_	−0.80	*
	ID_PROC_(1)	0.87	**
	P1	−0.06	ns
	P5	0.69	ns
	ΣRain	0.47	ns
	ΣT	0.76	*
	ID_PROC_(2)	0.66	NS
Reuler	DS_L5_	0.85	*
	T_nw_	−0.92	**
	ID_PROC_(1)	−0.40	ns
	P1	−0.83	ns
	P5	−0.70	ns
	ΣRain	−0.52	ns
	ΣT	0.72	*
	ID_PROC_(2)	0.83	ns

^a^ DS_L5_: Septoria leaf blotch severity on L5 at L3 emergence; T_nw_: number of days with negative air temperature during December 21-March 21; ID_PROC_(1): number of simulated potential daily infection events between GS 31 and GS 65 (simulations performed using PROCULTURE); P1 and P5: number of days with rainfall ≥ 1 mm and rainfall ≥ 5 mm between GS 31 and GS 65, respectively; Σrain: total rainfall between GS 31 and GS 65; ΣT: sum of mean air temperatures between GS 31 and GS 65; ID_PROC_(2): number of simulated potential daily infection events > 1 between GS 31 and GS 65 (simulations performed using PROCULTURE). ^b^ The *p*-value under the null hypothesis of zero correlation. Significance levels: *: *p* < 0.05; **: *p* < 0.01; ***: *p* < 0.001; and ns: *p* > 0.05.

## Data Availability

The datasets supporting this article are available from the corresponding author upon reasonable request.
